# 高分辨率熔解曲线法在*BIM*基因2, 903 bp片段插入/缺失检测中的应用

**DOI:** 10.3779/j.issn.1009-3419.2014.03.10

**Published:** 2014-03-20

**Authors:** 金晶 夏, 浩 白, 丽纹 熊, 蓉 李, 波 颜, 敏华 邵, 宝惠 韩

**Affiliations:** 200030 上海，上海交通大学医学院附属上海市胸科医院呼吸内科 Department of Respiratory Medicine, Shanghai Chest Hospital, School of Medicine, Shanghai Jiaotong University, Shanghai 200030, China

**Keywords:** 高分辨率熔解曲线法, *BIM*基因, 肺肿瘤, High resolution melting curve method, *BIM* gene, Lung neoplasms

## Abstract

**背景与目的:**

*BIM*基因编码的蛋白属于BCL2蛋白家族的成员，作为凋亡调控因子参与多种细胞活动。*BIM*基因上2, 903bp的插入缺失片段与非小细胞肺癌EGFR-TKI靶向用药的获得性耐药相关。建立并优化HRM方法有助于快速、准确地检测*BIM*基因2, 903 bp片段的缺失，为临床工作提供指导性的意见。本研究建立与稳定了HRM的检测方法，并在30例肺癌样本以及30例正常对照样本中检测*BIM*基因缺失情况。

**方法:**

设计、合成针对*BIM*基因片段缺失的引物，优化高分辨率熔解曲线（high resolution melting, HRM）分析方法。并选取部分样本通过普通PCR方法和直接测序法检测*BIM*基因缺失情况。野生型模板扩增产物其解链温度要高于缺失型产物的解链温度。野生型和缺失型产物的熔解曲线可见明显差异，相应的Tm值相差约2.5 ℃。

**结果:**

经过HRM基因突变分析方法，在30例肺癌样本中检测到1例为*BIM*基因纯合缺失型，7例为杂合缺失型，22例为野生型。在30例正常对照样本中检测，2例为杂合缺失型，28例为野生型。

**结论:**

本研究建立的对*BIM*基因片段缺失的高分辨率熔解曲线法是一种，灵敏、准确、快速、高通量的方法。

## Abstract (en)

**Background and objective:**

The aim of this study is to establish a HRM (high resolution melting curve) method for detection of deletion in human *BIM* gene and to detect this site deletion with the above method in 30 lung cancer samples and 30 normal samples.

**Methods:**

The primers for detection of BIM deletion were designed and synthesized. The HRM method for geng deletion was established. And select the part of samples to detect BIM delection by normal PCR and sequencing assay. The Tm value of wild type PCR products was higher than that of the deletion PCR products. The difference of the corresponding Tm value is 2.5 ℃.

**Results:**

By detection with HRM methods, 1 samples were confirmed to be mutant, 7 samples were confirmed to be heterozygous and the other 22 samples were all wild type in the lung cancer samples. 2 samples were confirmed to be heterozygous and the other 28 samples were all wild type in the normal samples.

**Conclusion:**

The HRM method for detection of BIM deletion established in this study is a sensitive, accurate, simple and high throughput method.

*BIM*基因是编码细胞凋亡基因BCL2蛋白家族中的一个成员，在细胞凋亡中发挥重要的调节作用^[1, 2]^。研究^[3]^表明*BIM*基因外显子3和外显子4之间存在的2, 903 bp的缺失会造成BIM亚型BH3结构缺乏表达。而这种缺乏表达会导致癌症患者对部分治疗药物有更强的耐药性。在癌症患者体内，这种删除与药物反应的持续时间相关联，并且能够被用来预测患者在没有疾病恶化时的存活时间。携带这种删除突变的那些患者的平均无疾病恶化存活期限为大约6个半月时间，而没有这种突变的那些患者则为将近12个月。因此BIM缺失的检测对于癌症患者有相当重要的意义。

在本研究中，建立了一种高分辨率熔解曲线法（high resolution melting, HRM）来检测BIM基因片段缺失在肺癌患者中的发生情况，并用传统PCR方法对HRM检测结果进行验证。结果表明，建立了一种高分辨率熔解曲线法对于*BIM*基因2, 903bp片段插入/缺失检测是一种灵敏、准确、快速、高通量的方法。

## 1 材料与方法

### 1.1 病例样本

共检测30例肺癌样本。30例正常对照样本。其中30例肺癌样本来自于上海市胸科医院2012年8月-2013年8月入院治疗的晚期非小细胞肺癌患者（Ⅲa期-Ⅳ期）。患者签署知情同意书后，抽取5 mL外周血，使用EDTA抗凝管进行保存。30例正常对照样本来自上海市徐汇区社会体检人群，俱签署知情同意。

### 1.2 DNA提取

基因组DNA采用浓盐法^[4]^进行抽提。取200 μL全血放入1.5 mL离心管内，加入裂解液STE（Tris-Hcl 10 mM, NaCl 20 mM, EDTA 1 mM）410 μL，10%SDS 90 μL，pk 5 μL在65℃消化15 h左右。充分消化后加入300 μL饱和NaCl轻摇3 min。加氯仿340 μL混匀后12, 000 rmp离心20 min。将上清液吸入另一离心管中，加异丙醇650 μL，4 ℃，12, 000 rpm离心16 min。倒掉异丙醇加500 μL 75%乙醇，4 ℃ 12, 000 rpm离心5 min，重复两次后烘干沉淀，加入70 μL Mili Q水溶解。提取的基因组DNA经Nanodrop定量，测定260/280值后，冻存备用。

### 1.3 引物设计与合成

根据GenBank中人类BIM基因序列（Gene ID: 10018），设计HRM上游引物：ATACCATCCAGCTCTGTCTTCATAG，HRM下游引物1：CCCAACCTCTGACAAGTGACC，HRM下游引物2：TTGGTGGGAATGTAAAATGGC。HRM引物设计原理见图 1。普通PCR上游引物：AATACCACAGAGGCCCACAG，普通PCR下游引物：GCCTGAAGGTGCTGAGAAAG。测序上游引物：CATAAATACCACAGAGGCCCACA GC，测序下游引物1：CCCAACCTCTGACAAGTG ACC，测序下游引物2：TTGGTGGGAATGTAAA ATGGC。

**1:**
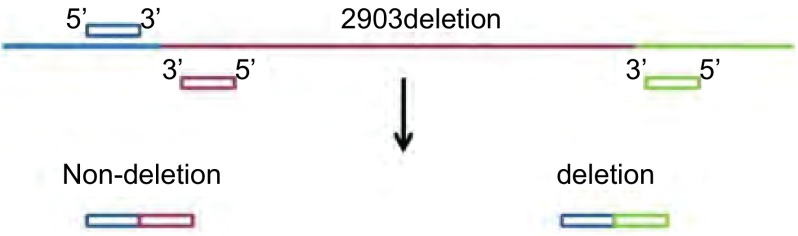
HRM检测*BIM*基因缺失引物设计原理 The principle of the HRM detection *BIM* gene deletion's primer design

### 1.4 HRM检测

PCR扩增和高通量熔解曲线分析在LightCycler^®^ 480 Ⅱ荧光定量PCR仪（Roche）上进行，采用384孔反应模块。试剂使用LightCycler^®^ 480 High Resolution Melting Master试剂盒，反应体系为10 μL，内含5 ng-10 ng基因组DNA、1×Master Mix、3.0 mmol/L MgCl_2_，引物浓度为0.2 μmol/L。

PCR扩增采用降落（touchdown）模式：95 ℃预变性10 min，95 ℃变性15 s，65 ℃-55 ℃退火（每循环下降0.5 ℃）15 s，72 ℃延伸20 s的程序进行20个循环。之后再按95 ℃变性15 s，55 ℃退火15 s，72 ℃延伸20 s的程序进行25个循环。扩增产物的熔解步骤在PCR循环结束后立即进行，程序为：升温至95 ℃ 15 s，然后降温至40 ℃ 1 min，再升温至65 ℃ 1 s。从65 ℃连续升温至95 ℃的过程中进行荧光收集（20次/℃）。最后，降温至40 ℃。高分辨率熔解曲线分析用LightCycler^®^ 480的Gene Scanning软件（1.5 version）进行。

## 2 结果

### 2.1 HRM检测方法的建立与优化

根据*BIM*基因缺失情况与序列信息，设计相应的引物。同一个反应中存在两对引物，可能扩增出两种不同的产物。为使两种产物都得到较好的扩增，采取了降落（touchdown）模式对模板进行扩增。缺失型模板的扩增产物与野生型模板扩增产物相比GC含量略低，解链温度也较低。分析结果显示，野生型和缺失型的模板所产生的熔解曲线可见明显差异，相应的Tm值相差约2.5 ℃（图 2）。在HRM的灵敏度检测中，分析评估不同BIM突变浓度DNA的结果（0%、1%、2%、5%、10%、25%、50%稀释和100%的突变体）。该HRM方法可以很容易区分突变含量为5%的样本（图 3），比普通PCR以及直接测序更高。

**2:**
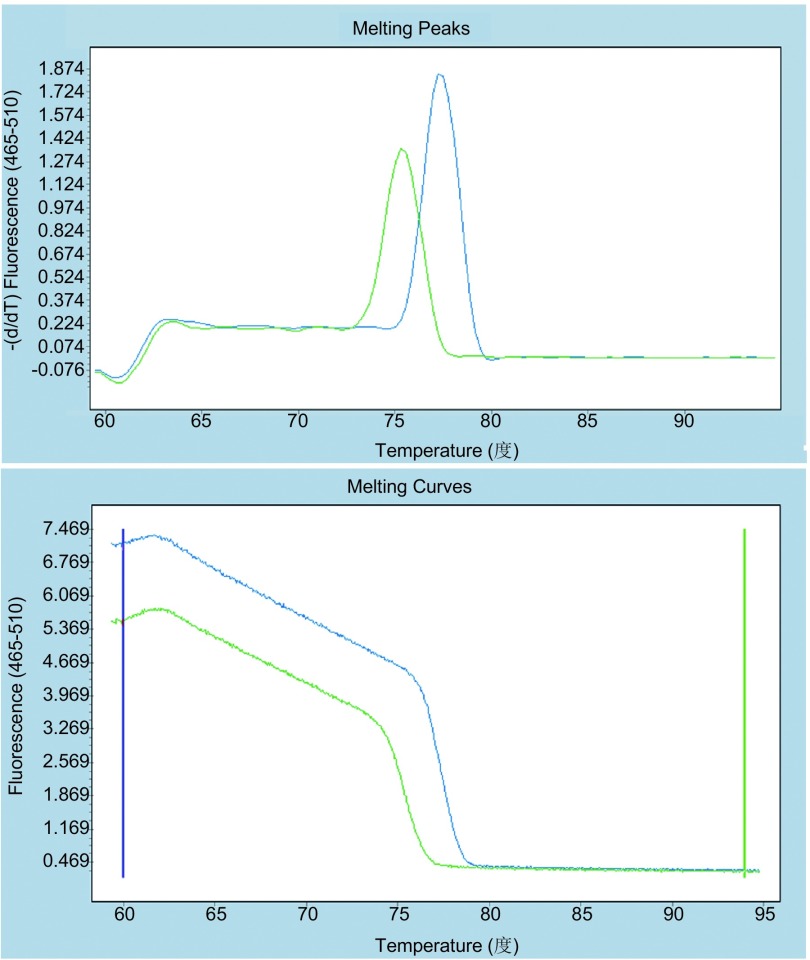
野生型比缺失型的解链温度要高。经变换后的熔解曲线峰值温度差值约为2.5 ℃，区分十分明显（——野生型——缺失型）。 The melting temperature of wild-type is higher than the deletion. Ater the data processing the significant difference of the melting curve peak temperature is about 2.5 ℃ (——wild type——deletion).

**3:**
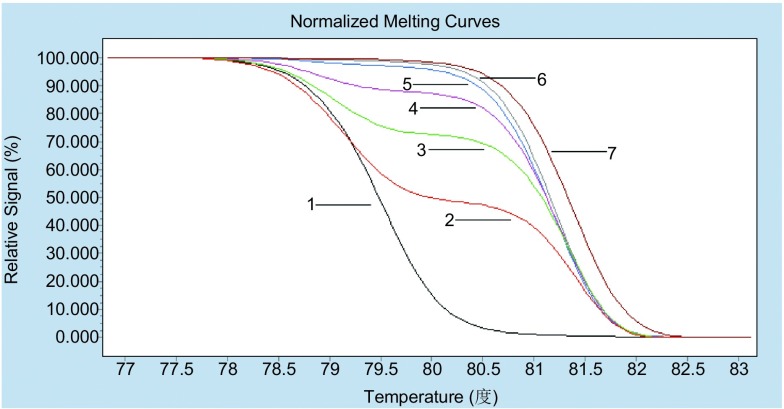
HRM检测BIM缺失灵敏度检测。1: 0%；2: 50%；3: 25%；4: 10%；5: 5%；6: 1%；7: 100%。 Sensitivity of the high resolution melting (HRM) analysis for detecting the BIM deletion. 1: 0% mutant; 2: 50% mutant; 3: 25% mutant; 4: 10% mutant; 5: 5% mutant; 6: 1% mutant; 7: 100%.

### 2.2 30例肺癌样本、30例正常对照样本*BIM*基因缺失检测结果

应用建立的HRM检测法，对30例肺癌样本，30例正常对照样本进行*BIM*基因缺失检测。在30例肺癌样本中发现纯合缺失型样本1例，杂合样本7例，其余均为野生型样本；在30例正常对照样本中发现杂合样本2例，其余均为野生型样本。从以上纯合缺失型、杂合缺失型、野生型样本中各取2例进行测序和普通PCR鉴定，普通PCR产物用2%琼脂糖凝胶电泳分析。

代表性的野生型、杂合缺失型和纯合缺失型的HRM与普通PCR检测结果如图 4所示，测序结果如图 5所示。图 4、5中a标本检测为野生型，b标本检测为杂合缺失型，c标本检测为纯合缺失型。

**4:**
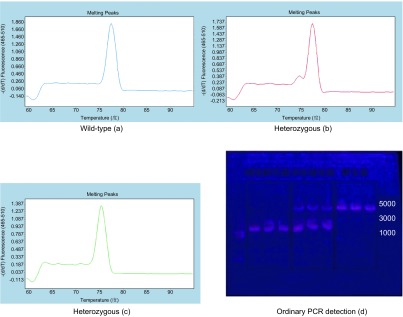
代表性野生型、杂合缺失型和纯合缺失型的HRM与普通PCR检测结果 The typical results of the the wild-type, heterozygous and homozygous' HRM and ordinary PCR detection

**5:**
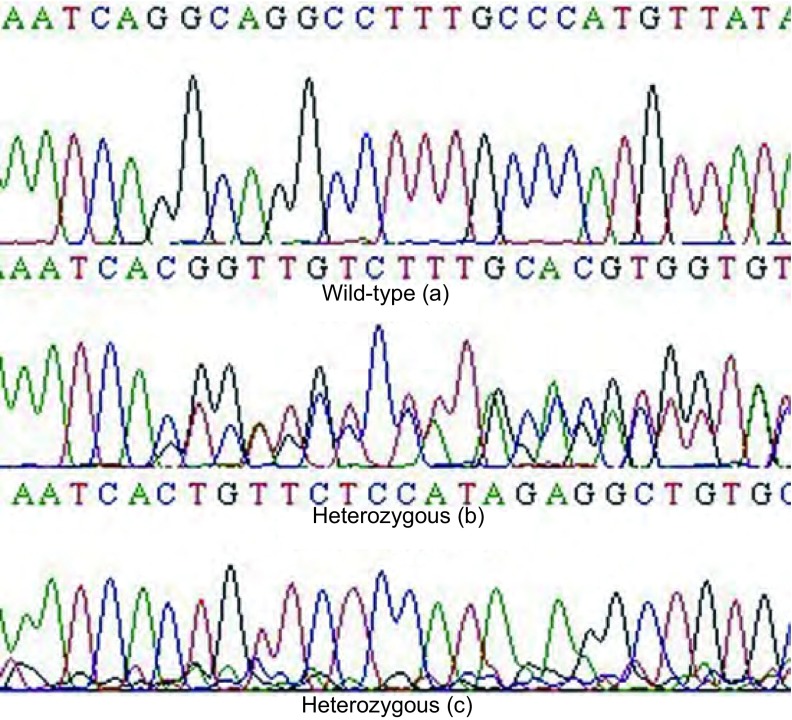
代表性野生型、杂合缺失型和纯合缺失型的测序结果 The typical results of the the wild-type, heterozygous and homozygous' sequencing detection

## 3 讨论

HRM可以检测扩增模板中的序列变化。该方法是通过检测与双链DNA结合的饱和荧光染料在变性升温过程中随着DNA解链时游离到体系中发生的荧光强度的减弱而实现的。与传统PCR以及测序方法相比较，此种方法可以在一步PCR反应中实现，无需延伸、无需进行电泳鉴定，有着快速、简便、重复性好、污染可能低的特性，而且只要是模板中有序列变化，都会使其熔解曲线发生变化，从而在高分辨率溶解曲线去得以体现，比传统PCR以及测序更灵敏^[5, 6]^。

HRM能检测模板中存在的任何序列变化。这使得该方法不但可以检测特定位点的突变，还能筛查整个扩增序列的变化。

*BIM*基因对细胞凋亡的调节起着极为重要的作用^[2, 7]^，在另外一项研究中发现*BIM*基因变异导致了某些患者无法受益于这些酪氨酸激酶抑制剂药物。药物耐受主要是*BIM*突变导致BIM蛋白生成受损。而使用BH3药物修复*BIM*基因功能就可能克服两种癌症类型的TKI耐受^[3]^。以上研究表明，*BIM*是个癌症耐药基因。Ng等^[3]^的研究中对*BIM*基因上2, 903 bp的indel的检测使用的方法为琼脂糖凝胶电泳方法，和本文阐述的HRM方法相比，凝胶电泳所需时间更长、检测需要开放式进行可能引入污染和交叉污染的机会，同时凝胶成像使用肉眼观察，如果PCR产物浓度较低会导致错误读取。相比，HRM方法使用荧光染料检测，灵敏度更高，原管检测避免了开管检测可能引入的污染机会，PCR反应完成后仅需要5 min-10 min进行熔解曲线检测，更方便快捷。本研究建立的对*BIM*基因2, 903 bp片段缺失的HRM是一种灵敏、准确、高效、快速、低污染的检测方法。
